# Winter oceanographic conditions predict summer bull kelp canopy cover in northern California

**DOI:** 10.1371/journal.pone.0267737

**Published:** 2022-05-05

**Authors:** Marisol García-Reyes, Sarah Ann Thompson, Laura Rogers-Bennett, William J. Sydeman

**Affiliations:** 1 Farallon Institute, Petaluma, California, United States of America; 2 Coastal Marine Science Institute, Karen C. Drayer, Wildlife Health Center, UC Davis, Bodega Marine Lab, Bodega Bay, California, United States of America; 3 California Department Fish and Wildlife, Bodega Marine Lab, Bodega Bay, California, United States of America; 4 Bodega Marine Lab, UC Davis, Bodega Bay, California, United States of America; Smithsonian - University of Washington, UNITED STATES

## Abstract

Bull kelp, *Nereocystis luetkeana*, is an iconic kelp forest species of the Northeast Pacific that provides a wide range of ecosystem services to coastal marine species and society. In northern California, U.S.A., *Nereocystis* abundance declined sharply in 2014 and has yet to recover. While abiotic and biotic stressors were present prior to 2014, the population collapse highlights the need for a better understanding of how environmental conditions impact *Nereocystis*. In this study, we used a newly-developed, satellite-based dataset of bull kelp abundance, proxied by canopy cover over 20 years, to test the hypothesis that winter oceanographic conditions determine summer *Nereocystis* canopy cover. For the years before the collapse (1991 through 2013), wintertime ocean conditions, synthesized in a Multivariate Ocean Climate Indicator (MOCI), were indeed a good predictor of summer *Nereocystis* canopy cover (R^2^ = 0.40 to 0.87). We attribute this relationship to the effects of upwelling and/or temperature on nutrient availability. South of Point Arena, California, winter ocean conditions had slightly lower explanatory power than north of Point Arena, also reflective of spring upwelling-driven nutrient entrainment. Results suggest that the *Nereocystis* gametophytes and/or early sporophytes are sensitive to winter oceanographic conditions. Furthermore, environmental conditions in winter 2014 could have been used to predict the *Nereocystis* collapse in summer 2014, and for kelp north of Point Arena, a further decline in 2015. Importantly, environmental models do not predict changes in kelp after 2015, suggesting biotic factors suppressed kelp recovery, most likely extreme sea urchin herbivory. Conditions during winter, a season that is often overlooked in studies of biophysical interactions, are useful for predicting summer *Nereocystis* kelp forest canopy cover, and will be useful in supporting kelp restoration actions in California and perhaps elsewhere in the world.

## Introduction

Canopy-forming kelps structure temperate and subpolar coastal ecosystems by providing habitat, food, and shelter to multitudes of fishes, invertebrates, seabirds, and marine mammals [1]. Due to their extensive spatial coverage and efficient CO_2_ uptake, kelp forests are also a key component in biogeochemical processes [2], which in turn provide a range of ecosystem services to people including direct exploitation, sustainable fisheries, recreational activities [3–5], and carbon sequestration [6]. In general, the highest rates of kelp growth occur in late winter and spring due to nutrient availability, and they are lowest in late summer and fall, when temperatures are higher and nutrient availability decreases [5]. Kelp are highly sensitive to their environment; often they are negatively impacted by higher temperatures [7], reduced nutrient availability [8], and strong wave and storm activity [9], as well as direct impact from human activities (see [5, 10] and references therein).

**Fig 1:**
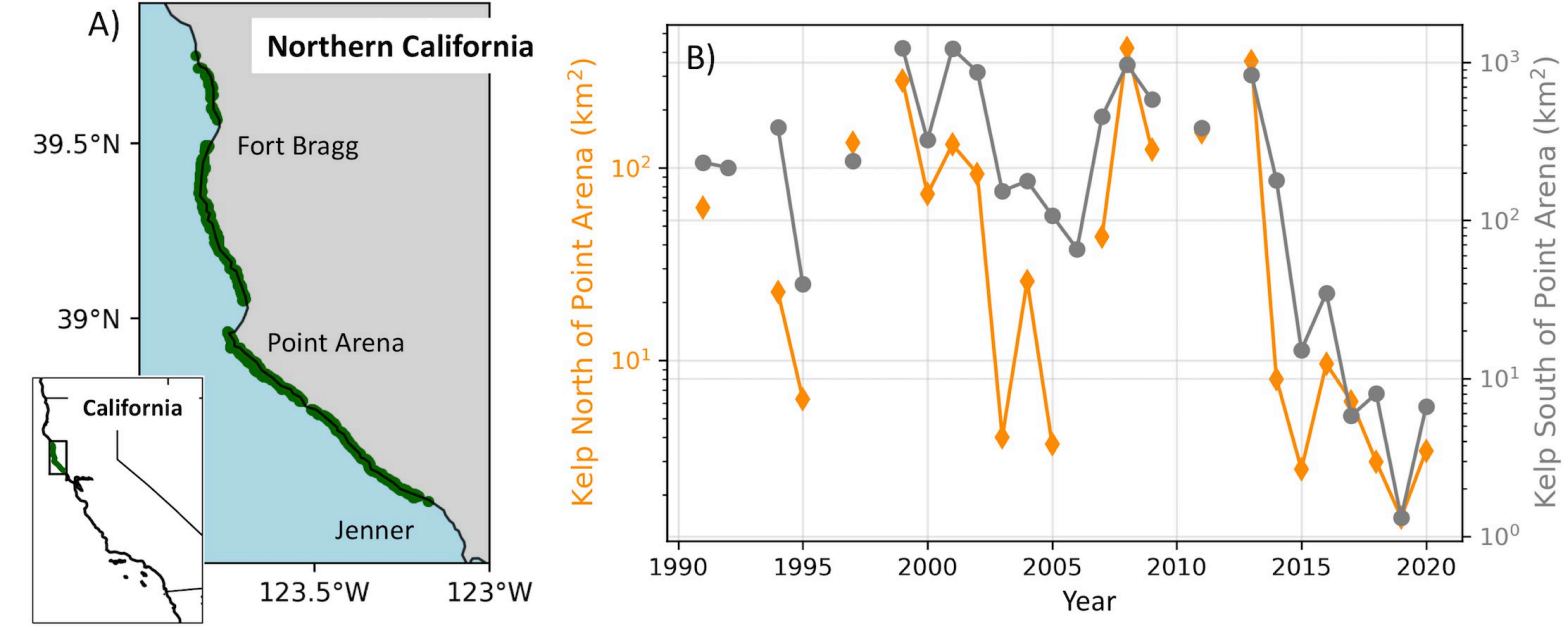
Bull Kelp geographical location and canopy extent. (A) Study region from 38–40°N on the northern California Coast, U.S.A. Green dots indicate kelp canopy data locations. Extensive sandy beaches are located north of Point Arena and Fort Bragg; no measurable kelp canopy occurs there. (B) Summer kelp canopy extent data (km^2^), 1991–2020, aggregated regionally for north (orange) and south (gray) of 39°N (Point Arena). Data derived from [34], freely available under CC BY 4.0 License at https://doi.org/10.6073/pasta/89b63c4b49b80fb839613e9d389d9902.

In the northeast Pacific, bull kelp (*Nereocystis luetkeana*) is the dominant canopy-forming species north of ~38°N, from northern California to Alaska. This annual kelp species is characterized by large floating pneumatocysts fastened by a single long stipe with its canopy residing mostly on the water’s surface. *Nereocystis* has historically formed large canopies along the central-northern California coast particularly between ~38 and 39.5°N and showed periodic short-term fluctuations in abundance (Fig 1) [11]. The *Nereocystis* annual life cycle involves the settlement of swimming zoospores produced by mature sporophytes, which develop into the over-wintering microscopic haploid gametophyte stage [12]. During winter, male and female gametes are released from the gametophytes, fertilize, and then develop into diploid zygotes, which grow into new sporophytes in the following spring [13]. Tiny sporophytes grow rapidly over the spring and can reach up to 25 m in length, forming the large adult kelp canopy visible on the surface in the summer. Sporulation occurs in late summer and fall when *Nereocystis* blades mature, but before storms and wave action dislodge the algae [14]. In lab experiments, *Nereocystis* has shown sensitivity to temperature, with an optimal growing temperature of ~11.9°C [15], and a reported thermal upper limit of 18–20°C [16, 17]. However, a recent experimental study suggested only partial reductions in blade growth rates at temperatures of 20°C [15]. Early life history stages (from germination to sporophyte development) have shown similar thermal tolerance below 18–20°C [17, 18]. There is limited understanding, however, on *Nereocystis* reproduction, dispersal, gametophyte sensitivity, and growth in relation to regional oceanography [19], particularly concerning the effect of seasonal oceanographic conditions on this annual canopy-forming kelp.

This lack of understanding of the relationship between survival of various life history stages and ocean conditions was made more apparent when the *Nereocystis* population of northern California collapsed in 2014 to less than 10% of its 2008 areal extent [20]. Further, this decline in kelp canopy has persisted (Fig 1), with some limited and spatially patchy recovery in 2021 (Rogers-Bennett personal observation). Multiple stressors were identified as present during the period of collapse [20] including: 1) a population decline of the sunflower sea star (*Pycnopodia helianthoides*), an important sea urchin predator [21], 2) grazing pressure from a rapid increase in purple sea urchin densities [20], and 3) potential thermal stress caused by an intense multi-year marine heatwave (MHW) that impacted a broad geographic region in the northeastern Pacific [22] and reached the California coast in summer 2014 [23]. Increased urchin density is a well-documented factor in kelp forest collapse around the globe [10, 24], yet in northern California, purple urchin densities in 2014 were not anomalously high (see [11]). Rogers-Bennett and Catton [20] as well as McPherson et al. [11] noted that kelp recovery has been hindered by the unprecedented increases in purple urchin densities in northern California.

The role that the 2014–2016 MHW [23] played on the *Nereocystis* decline is poorly understood, partly because long-term biophysical relationships between *Nereocystis* and the environment have not been adequately studied. Extreme temperatures are a known stressor for kelps in general [5]. However, sea surface temperatures during the 2014 MHW only sporadically reached magnitudes of 17°C around Point Arena, California (Fig 2 in [25]), which is below the documented *Nereocystis* thermal tolerance limit of 18–20°C [15–18]. Moreover, despite the fact that the MHW impacted the entire North American west coast [23], kelp forests only collapsed in some areas of California [21, 26, 27]. McPherson et al. [11] suggest that the high summer temperatures and low spring nutrient content in the ocean water may have been a key player in the 2014 *Nereocystis* collapse.

**Fig 2:**
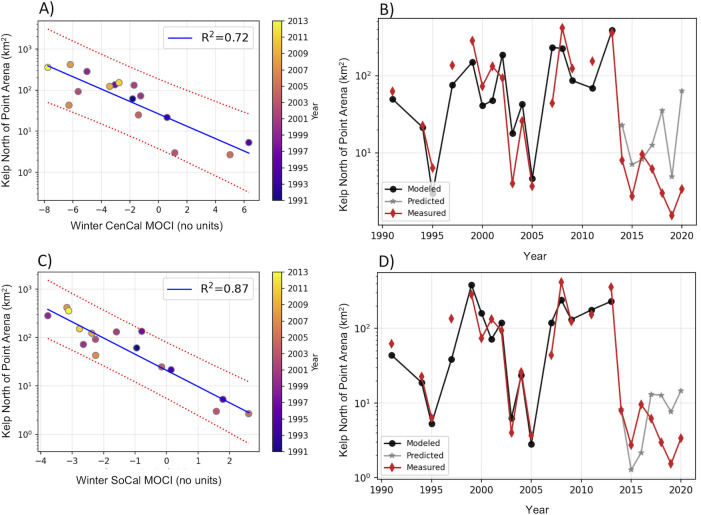
Linear regression between canopy coverage north of Point Arena and MOCI. (A) Linear regression between central California MOCI (CenCal MOCI) and kelp canopy extent north of Point Arena, 1991–2013, red-dotted lines indicate the confident intervals; R^2^ indicates the explained variance and the color indicates the year. (B) Time series of summer kelp canopy extent north of Point Arena: measured data are in red, modeled pre-collapse data are black, and predicted data for collapsed years are shown in grey asterisks (*). Modeled and predicted kelp uses the linear regression in (A). (C) Same as (A) but for winter southern California MOCI (SoCal MOCI). (D) Same as (B) but model and predicted data used the linear regression with southern California MOCI shown in (C). Regressions details are in Table 2.

Importantly, summer kelp abundance may be impacted by oceanographic conditions during earlier seasons, thereby affecting early life history stages. Of particular interest are wintertime conditions, which in the California Current are highly variable [28] and are known to play a large role in the productivity of the pelagic ecosystem in subsequent seasons [29, 30]. For example, groundfish growth and seabird survival in the central California Current are negatively impacted by warm winters and weak late-winter/early-spring upwelling [28, 31]. Winter ocean conditions may explain variation in *Nereocystis* canopy cover in this region, impacting early life history stages, but to date these ideas have yet to be investigated.

In this study, we use a new 20-year annual-scale dataset of kelp canopy cover to examine the effects and predictive capacity of seasonal oceanographic conditions on *Nereocystis* canopy cover in northern California. In particular, we test the hypothesis that winter oceanographic conditions determine summer *Nereocystis* canopy cover, and that conditions in winter 2014 played a role in the dramatic decline observed later that year. To test this hypothesis, we model the relationship between fall, winter, spring, and summer ocean conditions, synthesized by the Multivariate Ocean Climate Indicator (MOCI) [32, 33] on *Nereocystis* canopy cover variability in summer. We also examine potential mechanistic linkages by considering how temperature and upwelling-driven nutrient availability may affect variability in kelp abundance. Finally, we suggest that biophysical models may inform kelp restoration efforts in the future.

## Data and methods

The canopy data [34] is based on Landsat 5, 7, and 8 imagery providing the area (m^2^) covered by bull and giant kelp canopy along the coast. The spatial resolution is 30 x 30 m per pixel extending from Baja California Sur, Mexico, to Washington State, U.S.A., and it is resolved quarterly (seasonal) from March 1984 to December 2020. While kelp canopy cover measured in this way cannot be explicitly identified as *Nereocystis*, it is assumed that the majority of the observed canopy north of 38°N is indeed *Nereocystis* as it is the dominant, canopy-forming species in the region. In contrast, giant kelp (*Macrocystis pyrifera)* is dominant between San Francisco Bay and northern Baja California, but north of San Francisco, its presence is limited to small quantities in sheltered bays as it is more susceptible to wave forcing. Although the presence of perennial giant kelp (or other canopy-forming species) could lead to an overestimation of *Nereocystis* in our satellite imagery dataset, its extent is small in comparison, as illustrated by the difference in canopy cover between winter and summer/fall (S1 Fig).

We extracted and use *Nereocystis* data from summer (July–September), 1991–2020, for the coastal region between 38°N and 40°N, when the *Nereocystis* canopy has reached its annual peak in aerial extent (S1 Fig). Our study was also divided into two regions: north and south of Point Arena (39°N); *Nereocystis* canopy cover differs between these regions (S1 Fig). Importantly, Point Arena marks a division in oceanographic conditions due to a combination of topography and change in coastal orientation [35]. This results in the strongest upwelling in the California Current [36] downstream (south) of Point Arena, where upwelling plumes of cold and nutrient-rich water dominate ocean conditions [37, 38]. Coincidentally, a large stretch of sandy beach on the north side of Point Arena is devoid of kelp, producing a natural break in the kelp canopy data.

To estimate the total extent of the kelp canopy within a region required aggregating the coverage value of all pixels, however, we cannot distinguish between a pixel with no data due to obscuring cloud cover and a pixel with zero kelp cover. To ensure we did not count missing data as zeros we imposed thresholds of ‘available’ data to do these aggregations. First, we divided the region into bands of 0.1-degree latitude and summed the available data for each summer in each band. For each band, we calculated the number of pixels containing data each year and then the median number of pixels with data across all years in the data set. In any year, if a band had < 90% of the median number of pixels, it was assigned as a missing value. In this way, we accounted for areas with missing pixels due to data quality or cloud cover. Next, we summed the data in each region, north and south of Point Arena (referred to also as northern and southern kelp, respectively). For this we also imposed a threshold of only 10% of missing data, otherwise the summer was assigned a missing value. Years with missing data due to these gaps were 1993, 1996, 1998, 2010, and 2012 for both regions, and 1992 and 1996 for the northern region only.

To test that winter conditions influenced summer *Nereocystis* canopy, and also if winter is the only or most relevant season, we examined summer kelp co-variance with seasonal oceanographic conditions, lagged (fall, winter, and spring) and not-lagged (summer). We used the MOCI, which tracks the main mode of variability in ocean conditions along three regions (northern: north of 38°N, central: 34.5–38°N, and southern: south of 34.5°N) of the California coast [32, 33]. MOCI is calculated as the first principal component of sea and air surface temperature, sea level pressure, and alongshore winds from NOAA buoys over the continental shelf, the Bakun upwelling index [39], sea level from NOAA shore stations, and the climate oscillations: Multivariate El Niño Southern Oscillation (ENSO) Index (MEI)[40], Pacific Decadal Oscillation (PDO)[41], and North Pacific Gyre Oscillation index (NPGO)[42], see S2 Fig. Seasonal MOCI values used in the analyses were: fall (OND: October, November, and December; lagged for the year previous to the summer kelp canopy data (labeled fall-1)), winter (JFM: January, February, and March), spring (AMJ: April, May, and June), and summer (JAS: July, August, and September).

To gain insight into how ocean conditions influence kelp growth, we investigated two potential mechanisms: thermal stress and nutrient availability [5]. To test the effect of thermal stress, we used sea surface temperature (SST) data from NOAA buoy 46014 off Point Arena (labeled N14) and buoy 46013 south off Jenner (N13, see map in S2 Fig). Note that SST is already included in MOCI, but here we test a specific mechanism not possible by using MOCI alone. In this region, nutrients become available to the coastal areas through mixing and upwelling, and nutrient (nitrate) concentration has a tight inverse relationship with water temperature [36, 43]. We used two environmental variables to track nutrient availability for kelp: SST for overall nutrient content, and the Biologically Effective Upwelling Transport Index (BEUTI), an oceanographic index that tracks nutrient input at the coast driven by coastal upwelling [36]. We analyzed this index at 37°N, 39°N, and 41°N as it shows differences in values along the northern California coast associated with centers of upwelling. The monthly values of these indices were averaged seasonally to coincide with MOCI seasons. Time series of MOCI, SST, and BEUTI indices and data details can be found in S3–S10 Figs.

The shared period of kelp and environmental data was 1991–2020, which we divided into kelp pre-collapse (1991–2013) and post-collapse (2014–2020) periods. We investigated the relationships between summer kelp canopy cover and MOCI using univariate regression models, and then between kelp canopy and individual environmental variables using linear regressions; we assumed significance at p < 0.05 and used AIC values (Akaike Information Criterion) [44] to select the best models. We cross-validated each model and its predictability skills by running the selected model and removing each year at a time; average R^2^ for all “boot-strapped” models was taken as precision. Predictability errors were estimated in the same way: the model was run without one year at a time, and that year was predicted using that model. The error in prediction was calculated as absolute (measured—predicted)/measured, and expressed as a percentage. Finally, full pre-collapse models (1991–2013) were used to predict kelp values for 2014 onward. All analyses were performed in Python 3.7 using the scikit-learn package. Code is available in GitHub (https://github.com/farallon-institute/Garcia-Reyes_etal_BullKelp).

## Results

Kelp canopy south of Point Arena covered ~5x more area than north of Point Arena (Figs 1 and S1), and though interannual covariability between regions was substantial (⍴ = 0.87, p < 0.001, ~50% of shared variance), we considered these regions separately. No statistically significant linear trends were observed in summer kelp canopy time series from 1991 to 2013, although it is worth noting the reduced number of data points due to gaps and the large variability in canopy extent. During the pre-collapse period, however, environmental variables showed statistically significant trends in winter consistent with increasing upwelling and decreasing water temperature (S1 Table). There were not significant trends when the years 2014–2020 were included.

**Fig 3:**
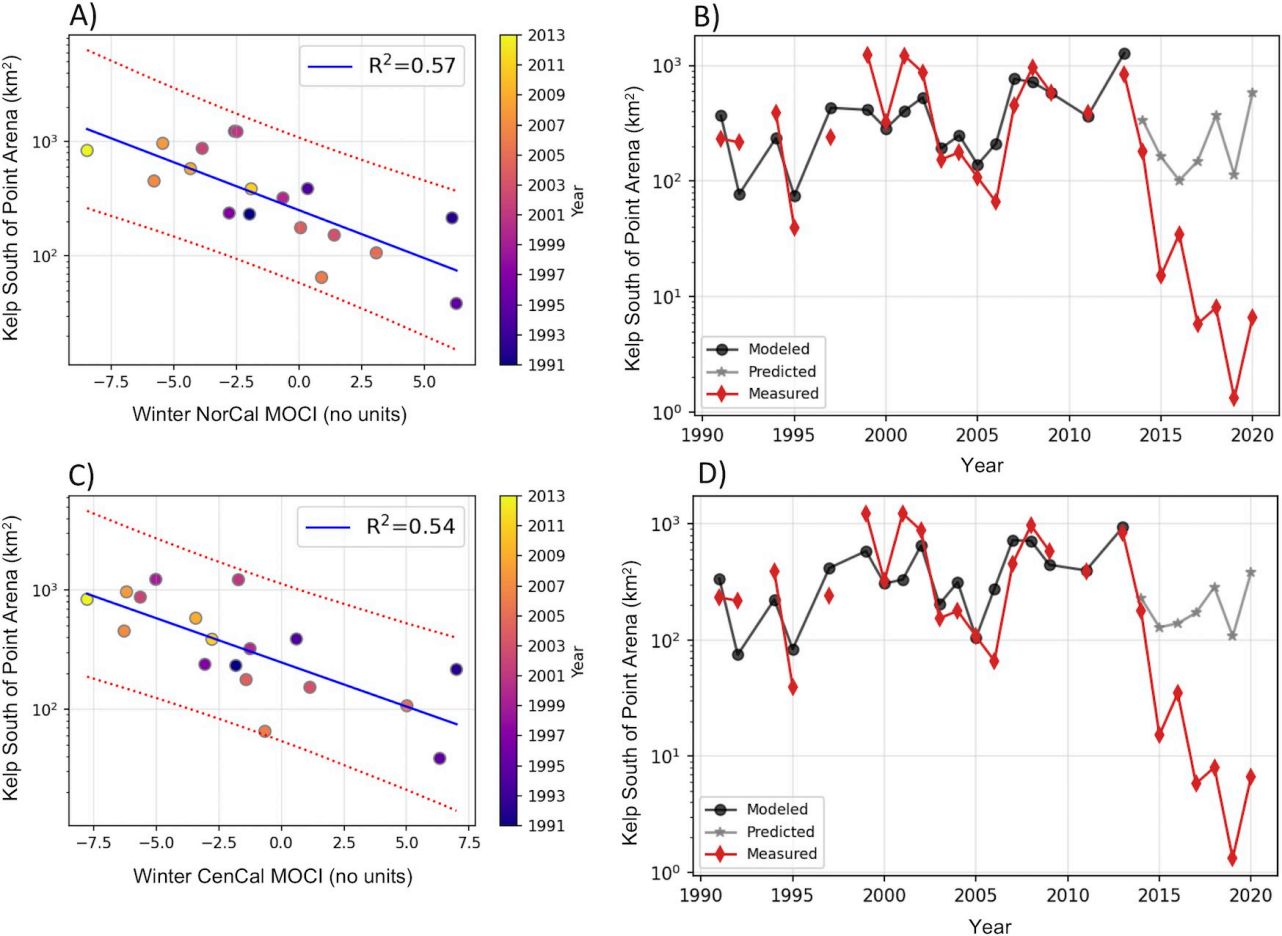
Linear regression between canopy coverage south of Point Arena and MOCI. Same as Fig 2 for regressions between kelp canopy south of Point Arena and northern California MOCI (NorCal MOCI; (A) and (B)) and central California MOCI, (CenCal MOCI; (C) and (D)).

Univariate linear regressions between seasonal MOCI and summer kelp canopy cover resulted in strong significant relationships for winter and spring MOCI, with R^2^ values ranging from 0.40 to 0.87 (Tables 1 and 2 shows regression details for selected models). Weak relationships were found with the previous year’s fall, and no significant relationships were established for the summer. The highest R^2^ values were found for winter southern and central California MOCI for kelp north of Point Arena (Table 1 and Fig 2) and for northern and central California MOCI for kelp south of Point Arena; surprisingly, the strongest correlation was northern kelp with southern California MOCI, but only slightly better. Spring relationships were slightly weaker than winter for kelp south of Point Arena and distinctly lower in the north, although spring R^2^ values were similar in magnitude for both regions. Univariate models for strong winter MOCI relationships are shown in Table 2 and illustrated in Figs 2 and 3 and S11. We did not include spring, since the correlation between winter and spring MOCI was high for the north and central California regions (S2 Table). Averaged R^2^ values from the cross-validation and predictability errors analysis for these models are reported in Tables 2 and 3, respectively. Cross-validated R^2^ values were consistent with, if not equal to, those of the model including all years (1991–2013). Average predictability error varied from 11% to 36%, and we note that there was large variability in the predictability for each year (8 to 52%). Also notable was that despite higher R^2^ values for models of kelp north of Point Arena, they had larger predictability errors than those for kelp south of Point Arena, especially with northern and central California MOCI.

**Table 1:** R^2^ values for linear regressions of kelp canopy cover.

	North of Point Arena				South of Point Arena			
	fall-1	winter	spring	summer	fall-1	winter	spring	summer
**NorCal MOCI**	-	0.67	0.48	-	-	0.57	0.51	-
**CenCal MOCI**	0.30	0.72	0.41	-	-	0.54	0.41	-
**SoCal MOCI**	0.40	0.87	-	-	-	0.40	-	-
BEUTI 41°N	-	-	0.37	-	-	0.25	0.38	-
BEUTI 39°N	-	0.30	0.40	-	-	0.46	0.43	-
BEUTI 37°N	-	0.48	0.40	-	-	0.48	0.51	-
SST N14	-	0.85	-	-	-	0.59	0.31	-
SST N13	-	0.83	0.29	-	-	0.59	0.40	-

R^2^ values for univariate linear regressions between kelp canopy cover north and south of Point Arena with same-year seasonal environmental variables (winter to summer) and previous-year variables (fall-1). Only significant (p<0.05) regressions are shown.

**Table 2:** Selected regression models of summer kelp canopy cover.

Equation	R^2^ (average R^2^ for cross-validation)	p-value	AIC
North Kelp = 3.32–0.36 * winter NorCal MOCI	0.67 (0.67)	<0.001	45.7
North Kelp = 3.27–0.35 * winter CenCal MOCI	0.72 (0.72)	<0.001	43.0
North Kelp = 3.04–0.77 * winter SoCal MOCI	0.87 (0.87)	< 0.001	30.7
North Kelp = 26.88–2.06 * winter SSTN14	0.85 (0.85)	< 0.001	32.8
South Kelp = 5.52–0.19 * winter NorCal MOCI	0.57 (0.57)	< 0.001	38.5
South Kelp = 5.51–0.17 * winter CenCal MOCI	0.54 (0.54)	<0.001	39.9
South Kelp = 16.82–0.98 * winter SSTN14	0.59 (0.59)	<0.001	37.9
South Kelp = 3.89 + 0.13 * spring BEUTI37N	0.51 (0.52)	<0.001	40.1

Equation, explained variance (R^2^), p-values of independent variables, and AIC values for selected regressions models. NorCal: northern California, CenCal: central California, SoCal: southern California, SSTN14: sea surface temperature at buoy N14, BEUTI37N: BEUTI index at 37°N.

**Table 3:** Predictive error of models for *Nereocystis*.

	Years	1991–2013	2014	2015	2016	2017	2018	2019	2020
**North of Point Arena**	**Winter NorCal MOCI**	36 (52)	39	100	1	71	183	572	158
	**Winter CenCal**	32 (44)	37	100	1	68	179	565	155
	**Winter SoCal MOCI**	15 (20)	1	76	66	41	133	380	120
**South of Point Arena**	**Winter NorCal MOCI**	11 (8)	12	87	30	185	184	1624	237
	**Winter CenCal MOCI**	11 (10)	5	79	39	193	171	1603	214

Error (precision) given in absolute percentages. The first data column indicates the predictability error for the model pre-collapse (1991–2013) including the average and standard deviation (in parentheses) values; the following columns show the predicted error for each year. NorCal: northern California, CenCal: central California, SoCal: southern California.

### Nutrient availability

**Fig 4:**
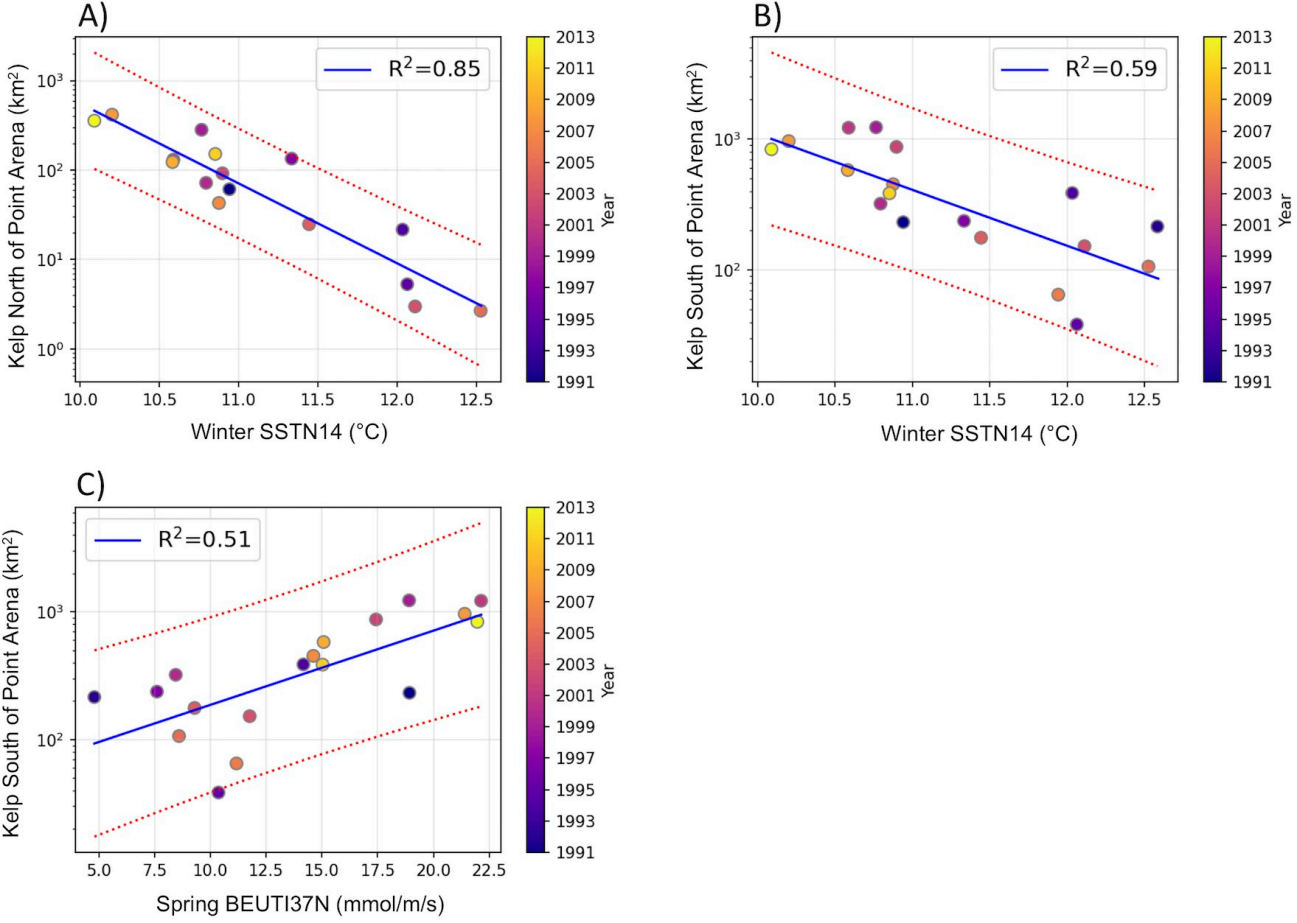
Linear regressions between canopy coverage and nutrient-related variables. Linear regression for the pre-collapse period 1991–2013, red-dotted lines indicate the confident intervals; R^2^ indicates the explained variance and the color indicates the year. (A) Summer kelp canopy extent north of Point Arena and winter SSTN14; summer kelp canopy extent south of Point Arena and (B) winter SSTN14, (C) spring BEUTI37N. Regressions details are in Table 2. SSTN14: sea surface temperature at buoy N14, BEUTI37N: BEUTI index at 37°N.

Linear regressions of summer kelp canopy with the environmental variables reflecting potential nutrient availability showed strong relationships with winter temperature, similar to those with winter MOCI, but relationships were weaker for BEUTI (Tables 1 and 2 and Fig 4). South of Point Arena, spring relationships were significant and comparable to those of winter MOCI for BEUTI at 37°N (Table 1). In both regions, spring relationships with BEUTI were better than those for SST. In the summer, these variables did not have significant relationships with *Nereocystis* abundance, indicating notably that kelp canopy coverage is not related to concurrent changes in temperature or upwelling-driven nutrient entrainment. Additionally, no significant relationships were found for previous year fall environmental conditions. Univariate models for strong relationships are shown in Table 2: winter SST at buoy N14 (similar to N13) was the strongest in both regions, and for south of Point Arena, also the model for spring BEUTI 37°N.

### Collapse and post-collapse years

*Nereocystis* canopy cover declined sharply beginning in summer 2014, declined further in 2015, and has shown little recovery as of 2020. It is worth noting that 2014 was not the lowest value of canopy extent in either region before 2015; 2014 was the 4th lowest year in the north, and 6th in the south. However, before 2014, kelp north of Point Arena showed resilience after occasional years with extremely low canopy cover years. In contrast, south of Point Arena, *Nereocystis* abundance has historically been relatively high and more stable before 2015.

To explore the role of oceanographic conditions on the 2014 *Nereocystis* population collapse and subsequent years’ lack of recovery, we used the selected MOCI-kelp regression models in Table 2 to predict kelp canopy extent values for years 2014–2020 (Table 3). For kelp north of Point Arena, the model including winter southern California MOCI predicted the 2014 collapse with very low error; for southern kelp, the winter central California MOCI model predicted the decrease observed in 2014. Other models had a larger predictive error. After the 2014 collapse (from 2015 on), the models had poor predictive powers and the predictive errors became much larger. We found that for northern kelp, models underestimated kelp in 2016, and also in 2015 for southern California MOCI, as the environmental conditions in those winters were highly unfavorable due to the marine heatwave. In the south, kelp models showed a further decrease in 2015 as well, but they largely overestimated the kelp canopy coverage. In 2017, models predicted a recovery that did not materialize in the field; a further decrease of kelp canopy was observed instead. By 2020, however, winter models and measured kelp extent for both regions showed a slight recovery, although modeled values still overestimated the measured values by at least an order of magnitude. This suggests that in the post-collapse time period, something other than oceanographic conditions impeded kelp recovery.

## Discussion

### *Nereocystis*’ relationship with oceanographic conditions

We investigated lagged relationships between northern California *Nereocystis* canopy cover during its peak season in summer and oceanographic conditions in the preceding seasons. We focused on the period 1991–2013, prior to the long-term decline in kelp abundance that began in 2014, to understand environmental drivers of abundance. As hypothesized, winter oceanographic conditions, tracked by MOCI, predicted kelp canopy in the summer. While there was some covariance in canopy variability in regions north and south of Point Arena (39°N), we found differences in the potential oceanographic mechanisms explaining kelp canopy extent. Remarkably, north of Point Arena, winter ocean conditions alone explained up to 87% of the interannual variability in kelp canopy cover. South of Point Arena, where kelp canopy is greater, winter conditions explained 57% of the variance. We surmised these relationships are driven by upwelling and temperature effects on nutrients.

*Nereocystis* is an annual species, and as such, all life history stages are likewise exposed to seasonal oceanographic conditions through the year [17]. We showed that in northern California, *Nereocystis* is most sensitive to oceanographic changes in winter, during its gametophyte life history stage. Of the variables considered, winter sea surface temperature (SSTN14), similar to MOCI, showed the highest R^2^ values, lowest AIC, and was able to predict summer canopy cover later in the year. Lethal impacts of warm temperatures on the *Nereocystis* gametophyte stage have been previously reported by Vadas [18] and Muth et al. [17] at 18–20°C. However, during the study period 1991–2020, seasonal or daily mean winter SST did not reach lethal limits (S6–S7 and S12 Figs), and indeed the maximum daily SST value encountered in winter was only 14.9°C. This suggests that mechanistically, thermal stress of gametophytes does not explain the correlation between ocean conditions and kelp canopy cover. However, temperature is also an indicator of nutrient availability. In the central California Current, temperature has a strong negative relationship with nitrate concentration [36, 43], and ocean water becomes nitrate limited at temperatures around 13°C. The importance of nutrients to kelp is well established [5, 45]. As spring approaches, nutrients are essential for gametogenesis and sporophyte production [46]. It is therefore likely that nutrient limitation associated with higher, but not lethal, temperatures (and perhaps weaker upwelling) could have led to the initial depletion of the *Nereocystis* population in northern California in early 2014. This interpretation is consistent with previous work that shows winter conditions have a large impact on the benthic and pelagic ecosystems of the central California Current (e.g., [28, 29, 33]).

Nutrient-related mechanisms help to explain the surprising result that the best predictor for kelp canopy extent north of Point Arena was the southern California MOCI, and not the central or northern MOCIs. All MOCIs include similar indicators: temperature, upwelling, and regional climate indices, but each regional MOCI represents a different ‘combination’ of variables according to the region’s variability. The central and northern California MOCI show greater influence from upwelling-related variables than does the southern California MOCI, for which temperature variables show greater influence [33]. The strong relationship of northern kelp with SSTN14 also supports the idea that the temperature-driven southern California MOCI is the best predictor of canopy cover.

As with the northern area, kelp canopy south of Point Arena shows a close association with winter conditions (in this case the northern California MOCI). There were also good relationships between kelp and winter SST, and the spring nutrient entrainment index (BEUTI37N). This suggests that in addition to winter temperatures, springtime upwelling-driven nutrient availability, as reflected by BEUTI, plays an important role in determining kelp canopy cover. This is not surprising as upwelling is the dominant oceanographic process in the region south of Point Arena due to the changes in coastal orientation there that leads to very strong upwelling-favorable winds [35, 36, 38]. The explained variance for spring BEUTI is slightly lower than winter SST, suggesting that while spring nutrient input to the coastal zone by upwelling is important to kelp growth, it is not as limiting as winter temperatures and associated winter nutrient availability. This is likely due to variable, yet prevalent entrainment of nutrients in spring due to upwelling (see S10 Fig), while during winter, the large SST variability could lead to very limited nutrient conditions (S6 and S7 Figs). Finally, the lack of significant relationships between summer kelp canopy extent and summer environmental variables suggests that by summer, *Nereocystis* abundance as measured by canopy cover has already been set by previous oceanographic conditions.

### *Nereocystis* canopy predictability

We established that oceanographic conditions in winter predict summer *Nereocystis* canopy cover from 1991–2013, with precision (predictability error) of 11 to 36% (Table 3). We then examined how ocean conditions predicted the collapse of the kelp forest in 2014, and particularly 2015, which was unprecedented in the observational record. The work presented here shows that regional ocean conditions in winter 2014, particularly temperature, were strongly unfavorable and could have been sufficient to cause the decline in kelp canopy extent observed by summer 2014, although more work is needed to elucidate the mechanism. McPherson et al. [11] also suggested that spring and summer environmental conditions have limited capability (explaining 28.3% of the variance) predicting the *Nereocystis* decline in 2014.

The warm conditions of the marine heatwave in winter 2015 likely contributed to the lack of kelp recovery. The low kelp canopy extent observed in 2015 north of Point Arena was accurately predicted by the winter environmental conditions. However, south of Point Arena, larger kelp canopy extent was predicted by winter conditions than was observed (Fig 3). This indicates that oceanographic conditions probably were not the only cause of continued decline in 2015 south of Point Arena. After 2015, as we have shown, ocean conditions no longer predicted kelp canopy cover (Fig 3). The models predicted a modest recovery of canopy extent beginning in 2017 as the MHW abated [23], but observed canopy actually declined further. In addition to the intense herbivory by large numbers of sea urchins, it is possible that cumulative conditions (i.e., 2+ years of unfavorable winter warming) could have had additional negative impacts on the kelp population, as these persisting poor conditions extended beyond the full *Nereocystis* annual life history cycle. Such an extended time of poor environmental conditions was unprecedented. It is worth noting that 2018–2020 changes in observed kelp canopy mirror the changes predicted by the models (declining in 2019 and increasing in 2020), although at a much-dampened scale due to increased herbivory. This suggests that *Nereocystis* is responding to a certain extent to winter oceanographic conditions. Grazing pressure due to increased sea urchin population that established while *Nereocystis* was decimated is the most likely explanation for the lack of kelp recovery [11, 20]. Further modeling efforts that include biotic factors are needed to help predict kelp extent in the post-collapse sea urchin barrens period. The models presented here, however, show the importance of oceanographic conditions to *Nereocystis* in the early life history stages.

### Implications for restoration

Kelp restoration planning would benefit from knowing which regions and years are best for restoration. Spatial decisions about where to conduct kelp restoration might also be improved by fine scale temperature information to aid in selecting cold water “climate refugia” [47]. Knowing which years are predicted to be favorable for the early life history stages of kelps so that various kelp restoration measures could be enacted with a higher likelihood of success. If oceanographic conditions signal a poor kelp year then the expense and effort of restoration measures, such as sea urchin control, might best be spent in future years with environmental conditions more favorable for kelp. Likewise, kelp restoration methods such as setting out twine lines that have been seeded with kelp [48] or green gravel seeding methods [49] would be best done in years when the oceanographic conditions are favorable for young kelp. In poor years, when warm winters are expected for the California coasts, proactive steps to protect and conserve *Nereocystis* could be taken. There is potential for successful restoration of local *Nereocystis* populations using an informed application of seeding and urchin control methods in careful consideration of environmental conditions. Our work highlights the need to consider complex oceanographic factors in different regions at seasonal scales to inform restoration and conservation efforts. Looking ahead, we suggest that winter MOCI may be particularly useful for predicting good years for kelp recruitment and survival in northern California, and could also be used to further investigate the influence of ocean conditions on other kelp such as *Macrocystis* and canopy forming kelps worldwide.

## Supporting information

S1 Fig Average canopy extent by season by region. Lines indicate the standard deviation around the mean canopy extent.
(PNG)

S2 Fig Location of regions and data included in MOCI. MOCI (Multivariate Ocean Oscillation Indicator) is a synthesized indicator of main variability mode of oceanographic conditions in southern, central and northern California, found at http://www.faralloninstitute.org/moci.
(PNG)

S3 Fig Northern California MOCI. Time series of winter, spring summer and fall values of Northern California MOCI (NorCal MOCI). Note that fall is not lagged in this and following plots as it is on the analysis with kelp canopy.
(PNG)

S4 Fig Central California MOCI. Time series of winter, spring summer and fall values of Central California MOCI (CenCal MOCI).
(PNG)

S5 Fig Southern California MOCI. Time series of winter, spring, summer and fall values of Southern California MOCI (SoCal MOCI).
(PNG)

S6 Fig Sea Surface Temperature at buoy N14. Time series of winter, spring, summer and fall averages of SST for buoy 46014 (SSTN14), located at 39.23°N 123.97°W. Data from https://www.ndbc.noaa.gov/. Gaps in the data have been filled using reanalysis data from the NOAA’s Optimal Interpolation SST dataset and neighboring buoys.
(PNG)

S7 Fig Sea Surface Temperature at buoy N13. Time series of winter, spring, summer and fall averages of SST for buoy 46013 (SSTN13), located at 38.25°N 123.30°W. Data from https://www.ndbc.noaa.gov/. Gaps in the data have been filled using reanalysis data from the NOAA’s Optimal Interpolation SST dataset and neighboring buoys.
(PNG)

S8 Fig BEUTI at 41°N. Time series of winter, spring, summer and fall averages of Biologically Effective Upwelling Transport Index (BEUTI, indicator of nutrients influx to the surface layer, integrating upwelling and temperature) at 41°N. Data from: https://oceanview.pfeg.noaa.gov/products/upwelling/cutibeuti.
(PNG)

S9 Fig BEUTI at 39°N. Time series of winter, spring, summer and fall averages of Biologically Effective Upwelling Transport Index (BEUTI, indicator of nutrients influx to the surface layer, integrating upwelling and temperature) at 39°N. Data from: https://oceanview.pfeg.noaa.gov/products/upwelling/cutibeuti.
(PNG)

S10 Fig BEUTI at 37°N. Time series of winter, spring, summer and fall averages of Biologically Effective Upwelling Transport Index (BEUTI, indicator of nutrients influx to the surface layer, integrating upwelling and temperature) at 37°N. Data from: https://oceanview.pfeg.noaa.gov/products/upwelling/cutibeuti.
(PNG)

S11 Fig Linear regression between summer canopy coverage south of Point Arena and winter northern California MOCI. (A) Linear regression and scatter plot between winter northern California MOCI (NorCal MOCI) and kelp canopy extent south of Point Arena, 1991–2013, red-dotted lines indicate the confident intervals; R^2^ indicates the explained variance and the color indicates the year (B) Time series of summer kelp canopy extent south of Point Arena: measured data are in red, modeled pre-collapse data are black, and predicted data for collapsed years are shown in grey asterisks (*). Modeled and predicted kelp uses the linear regression in (A).
(PNG)

S12 Fig Histogram of daily SST for N14. Daily SST values during winter (January-March) for buoy N14 for the period of study 1991–2020.
(PNG)

S1 Table Linear trends for environmental variables in winter. Only significant p<0.05 values shown (except BEUTI at 39°N), comparing two periods: pre-collapse (1991–2013), and entire period (1991–2020). Color indicates the sign of the trend.
(PDF)

S2 Table Ranked correlations of environmental variables between seasons. 1 season lag in the first three columns and 2 seasons lag in the last column. Only statistically significant correlations p<0.05 are shown.
(PDF)
