# Supplementary figures and images for: Winter oceanographic conditions predict summer bull kelp canopy cover in northern California
Source: PLoS One. 2022 May 5;17(5):e0267737. doi: 10.1371/journal.pone.0267737 (PMC9070938; doi:10.1371/journal.pone.0267737)

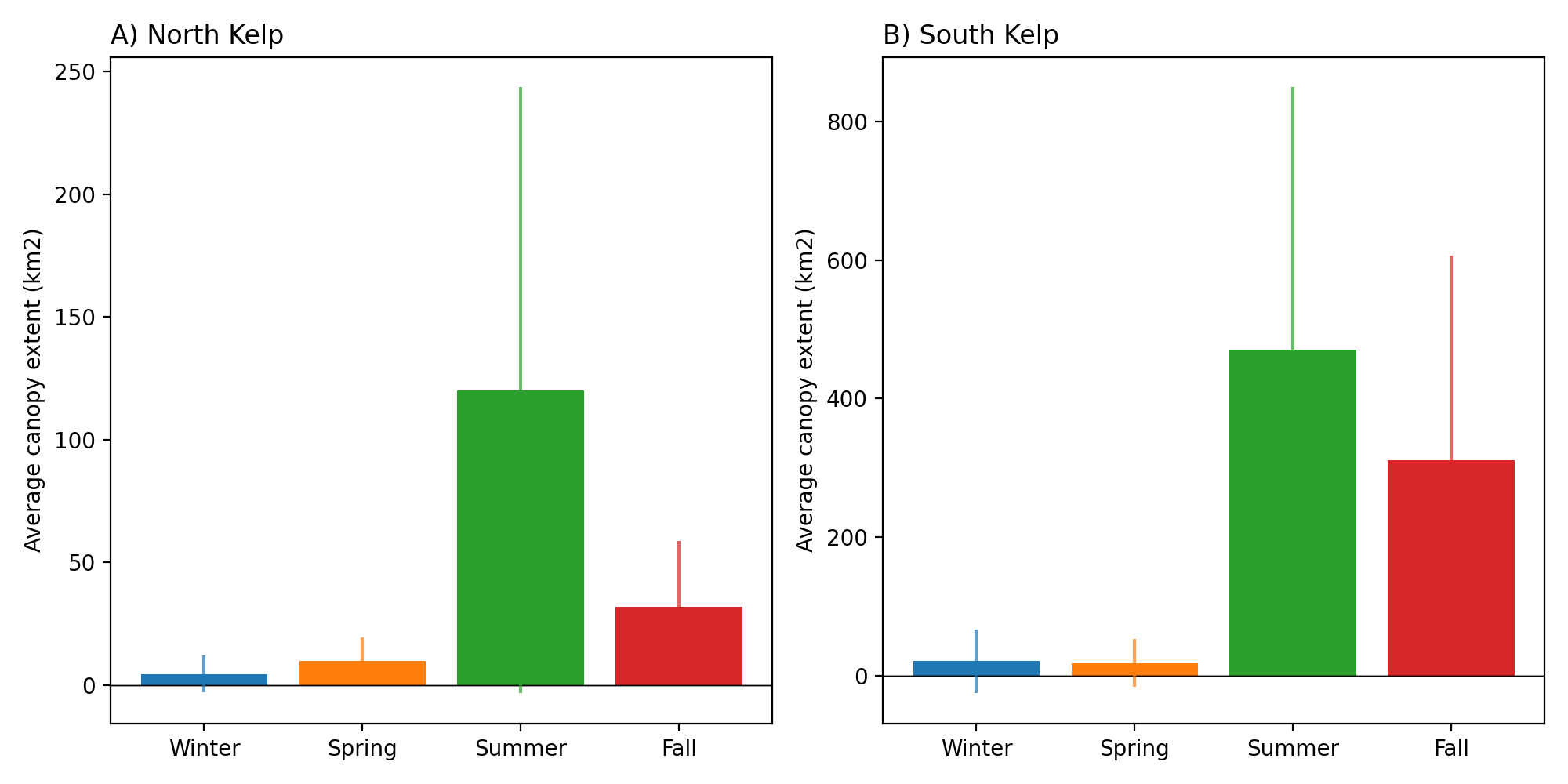

Supplement: S1 Fig — Lines indicate the standard deviation around the mean canopy extent. (PNG) [file pone.0267737.s001.png]

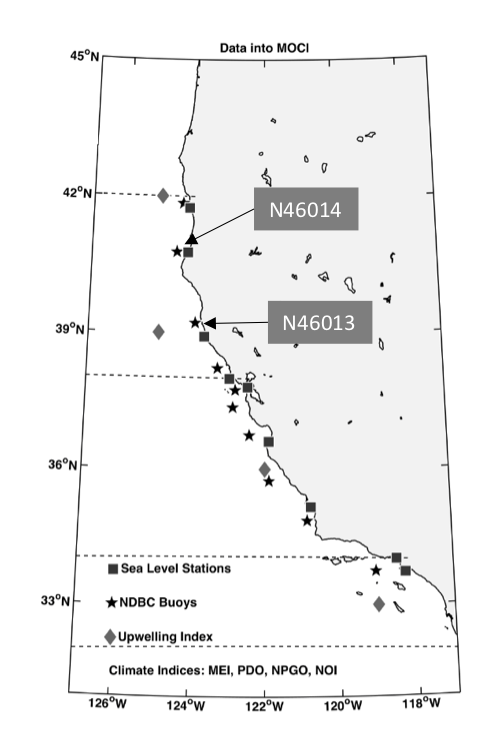

Supplement: S2 Fig — MOCI (Multivariate Ocean Oscillation Indicator) is a synthesized indicator of main variability mode of oceanographic conditions in southern, central and northern California, found at http://www.faralloninstitute.org/moci. (PNG) [file pone.0267737.s002.png]

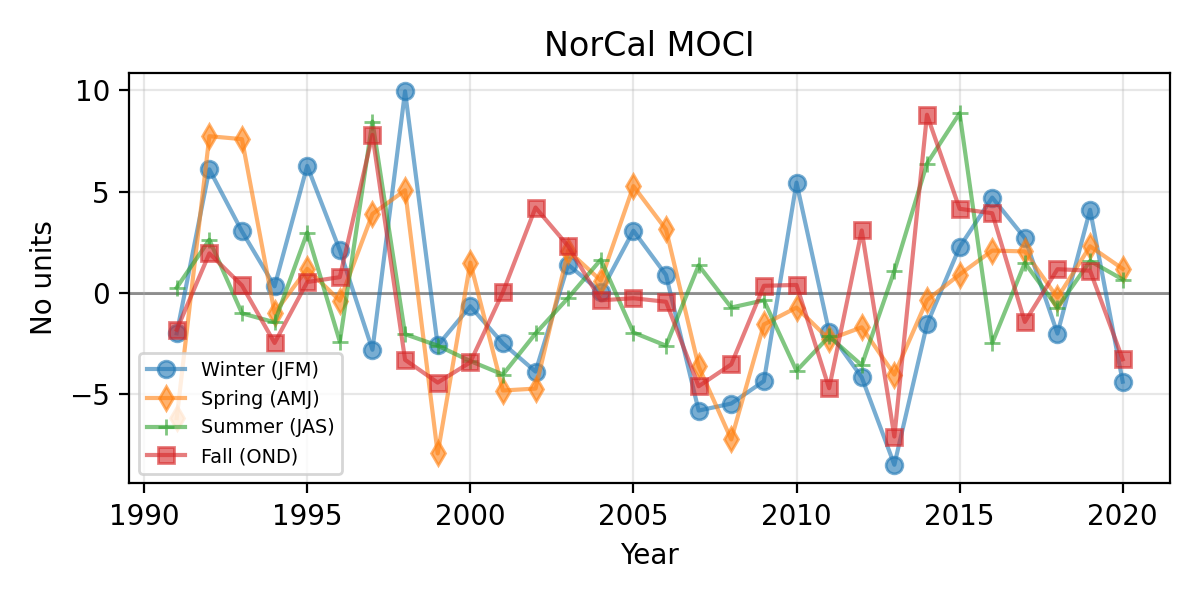

Supplement: S3 Fig — Time series of winter, spring summer and fall values of Northern California MOCI (NorCal MOCI). Note that fall is not lagged in this and following plots as it is on the analysis with kelp canopy. (PNG) [file pone.0267737.s003.png]

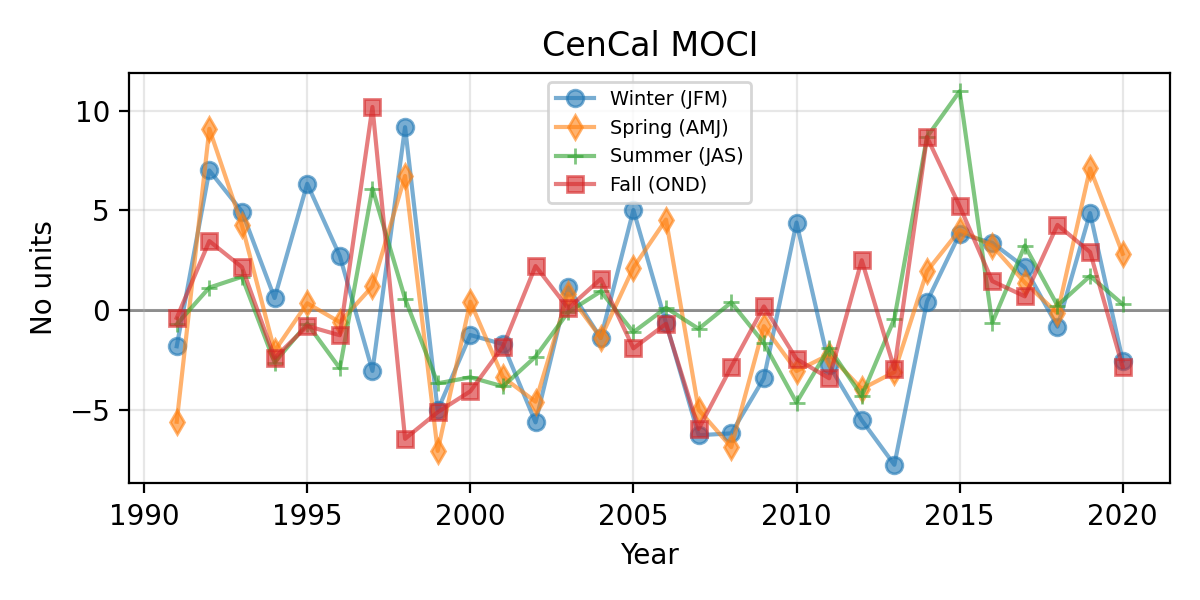

Supplement: S4 Fig — Time series of winter, spring summer and fall values of Central California MOCI (CenCal MOCI). (PNG) [file pone.0267737.s004.png]

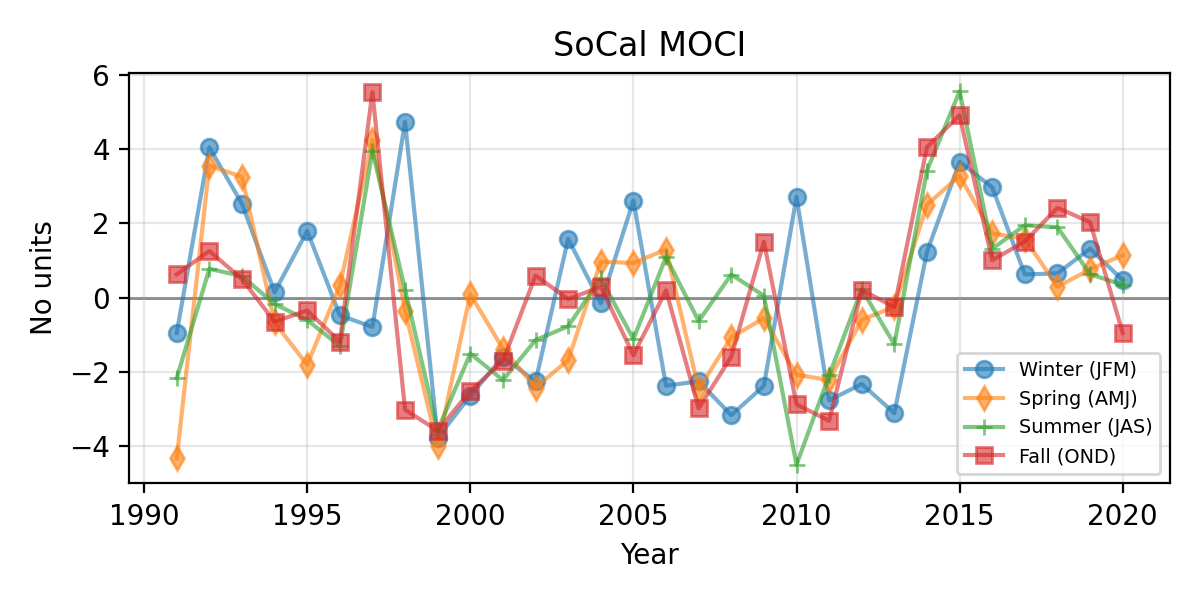

Supplement: S5 Fig — Time series of winter, spring, summer and fall values of Southern California MOCI (SoCal MOCI). (PNG) [file pone.0267737.s005.png]

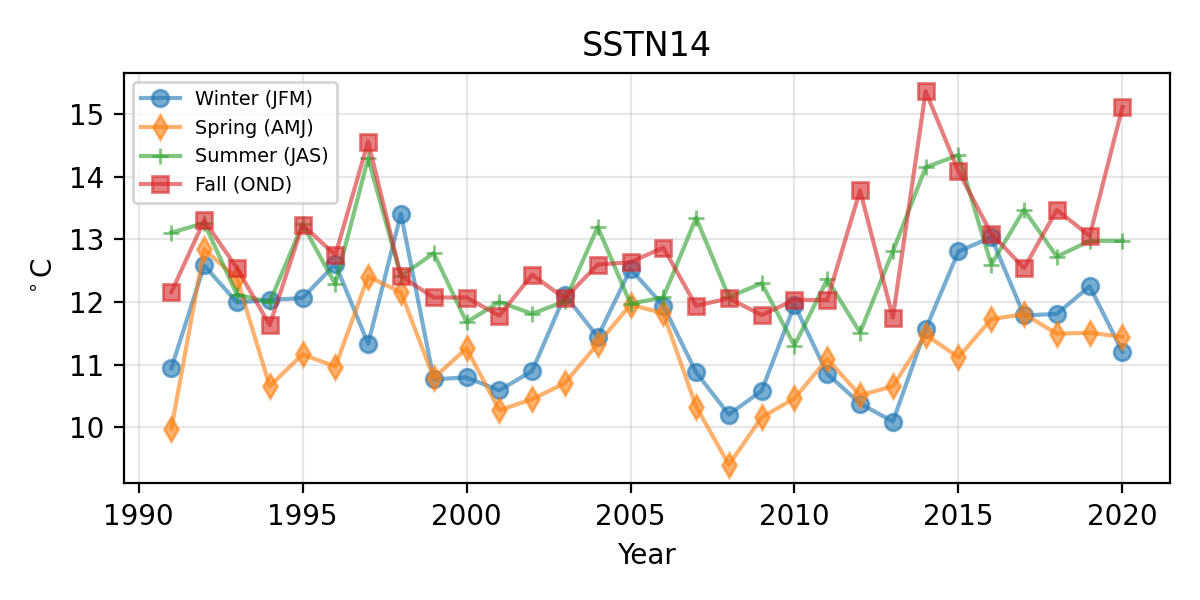

Supplement: S6 Fig — Time series of winter, spring, summer and fall averages of SST for buoy 46014 (SSTN14), located at 39.23°N 123.97°W. Data from https://www.ndbc.noaa.gov/. Gaps in the data have been filled using reanalysis data from the NOAA’s Optimal Interpolation SST dataset and neighboring buoys. (PNG) [file pone.0267737.s006.png]

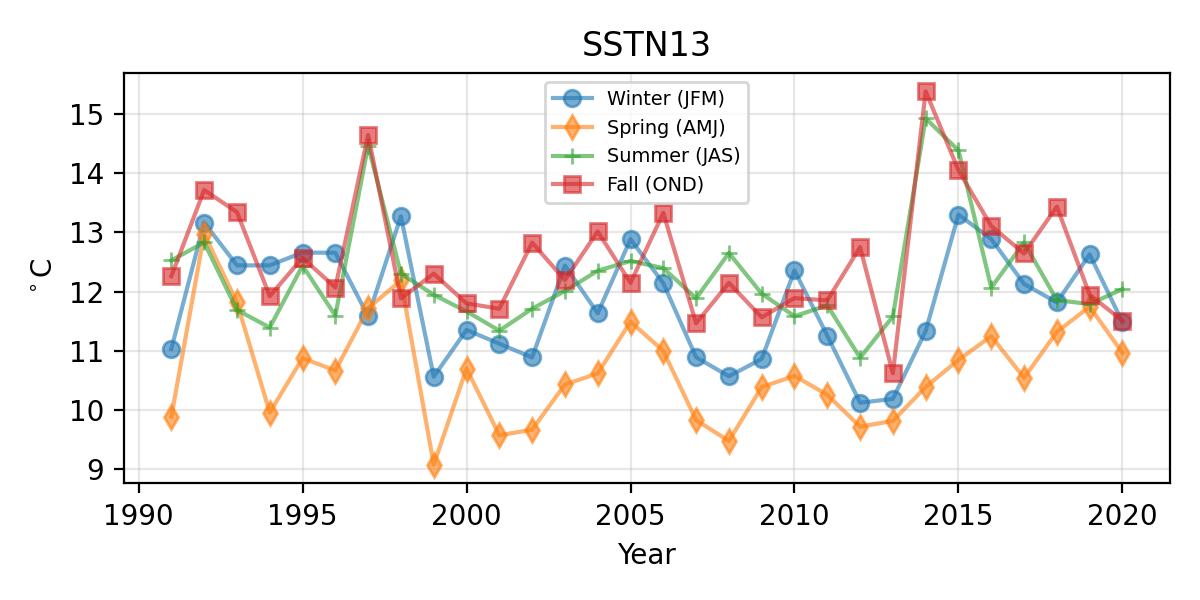

Supplement: S7 Fig — Time series of winter, spring, summer and fall averages of SST for buoy 46013 (SSTN13), located at 38.25°N 123.30°W. Data from https://www.ndbc.noaa.gov/. Gaps in the data have been filled using reanalysis data from the NOAA’s Optimal Interpolation SST dataset and neighboring buoys. (PNG) [file pone.0267737.s007.png]

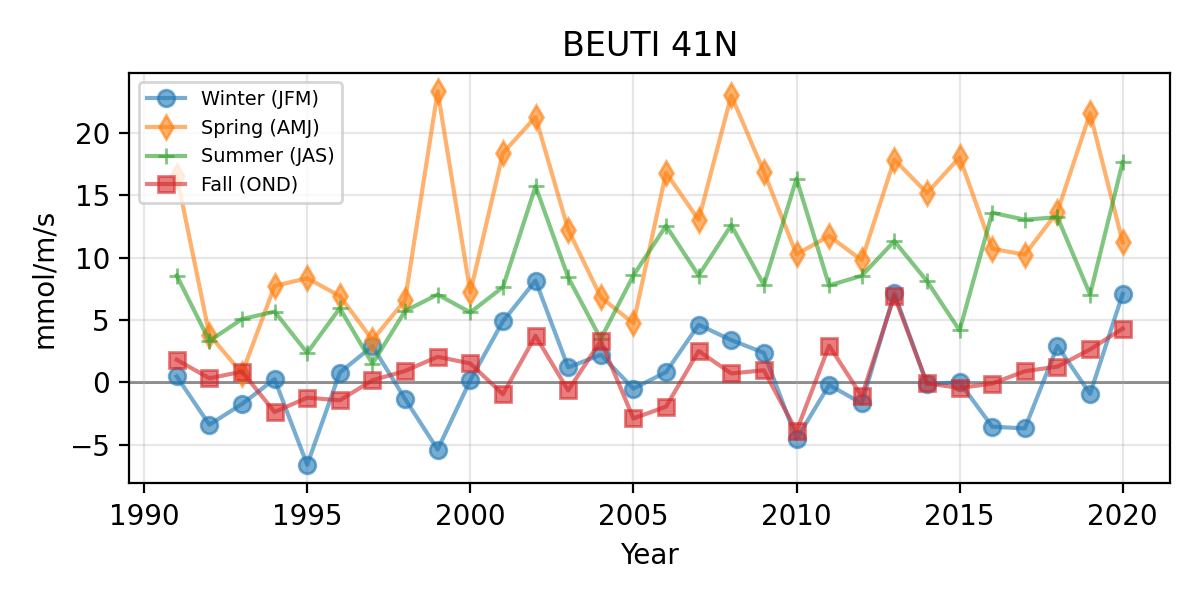

Supplement: S8 Fig — Time series of winter, spring, summer and fall averages of Biologically Effective Upwelling Transport Index (BEUTI, indicator of nutrients influx to the surface layer, integrating upwelling and temperature) at 41°N. Data from: https://oceanview.pfeg.noaa.gov/products/upwelling/cutibeuti. (PNG) [file pone.0267737.s008.png]

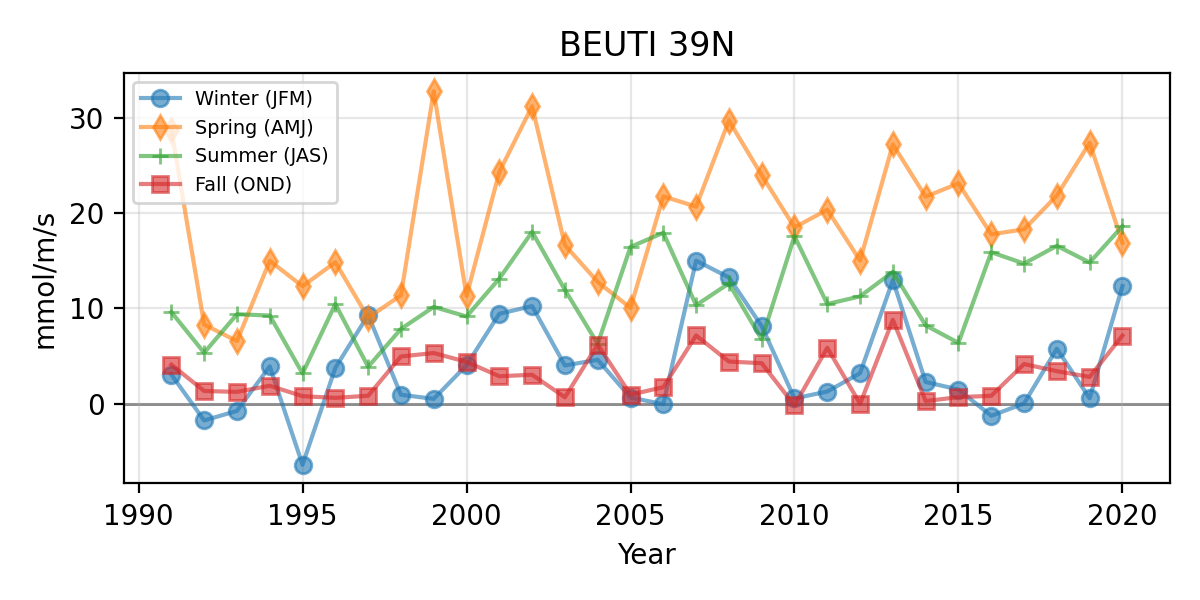

Supplement: S9 Fig — Time series of winter, spring, summer and fall averages of Biologically Effective Upwelling Transport Index (BEUTI, indicator of nutrients influx to the surface layer, integrating upwelling and temperature) at 39°N. Data from: https://oceanview.pfeg.noaa.gov/products/upwelling/cutibeuti. (PNG) [file pone.0267737.s009.png]

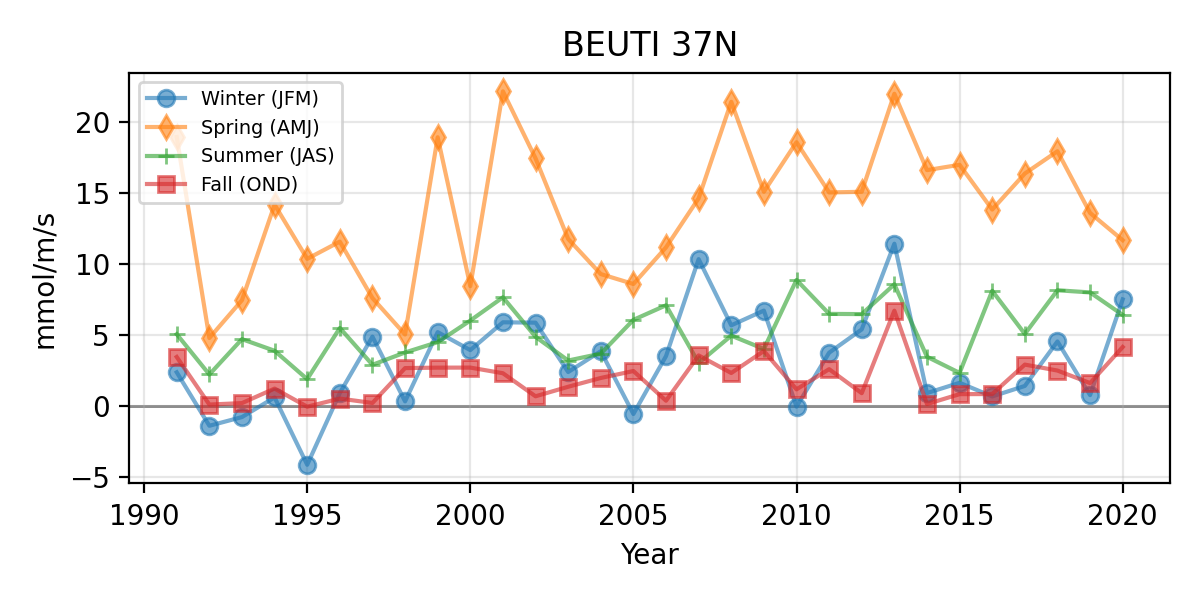

Supplement: S10 Fig — Time series of winter, spring, summer and fall averages of Biologically Effective Upwelling Transport Index (BEUTI, indicator of nutrients influx to the surface layer, integrating upwelling and temperature) at 37°N. Data from: https://oceanview.pfeg.noaa.gov/products/upwelling/cutibeuti. (PNG) [file pone.0267737.s010.png]

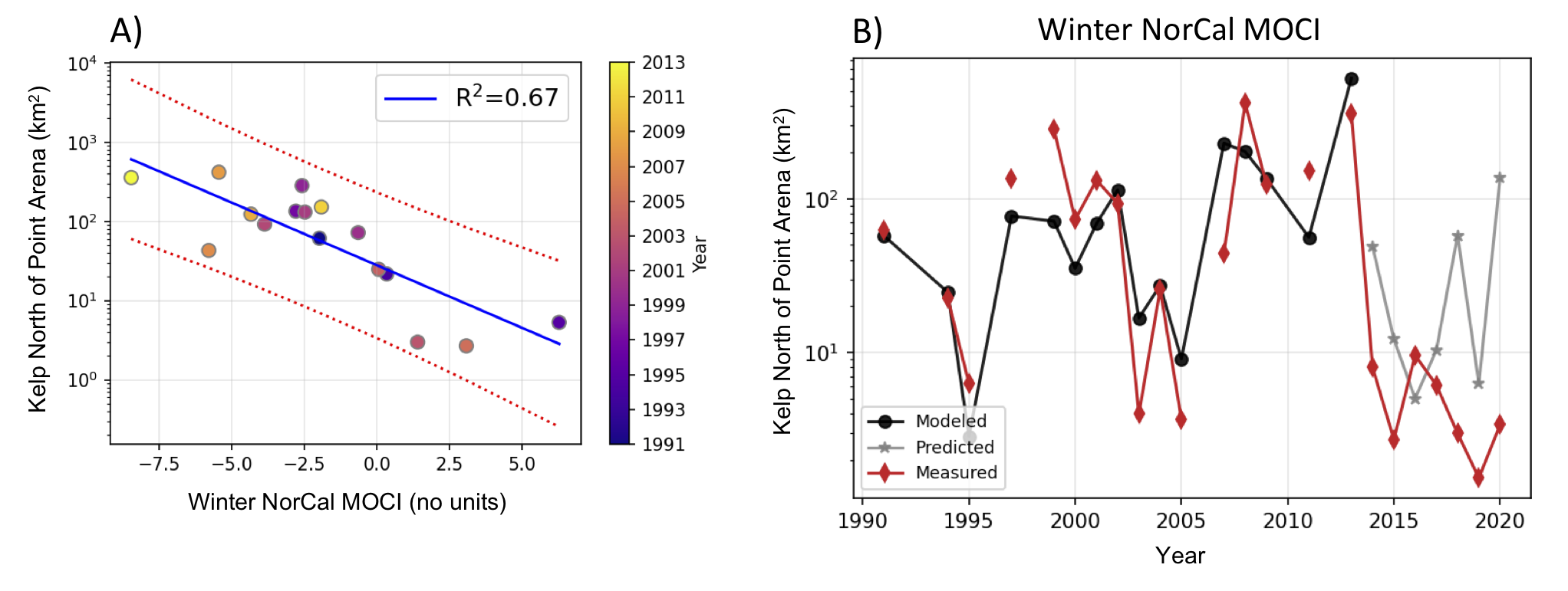

Supplement: S11 Fig — (A) Linear regression and scatter plot between winter northern California MOCI (NorCal MOCI) and kelp canopy extent south of Point Arena, 1991–2013, red-dotted lines indicate the confident intervals; R2 indicates the explained variance and the color indicates the year (B) Time series of summer kelp canopy extent south of Point Arena: measured data are in red, modeled pre-collapse data are black, and predicted data for collapsed years are shown in grey asterisks (*). Modeled and predicted kelp uses the linear regression in (A). (PNG) [file pone.0267737.s011.png]

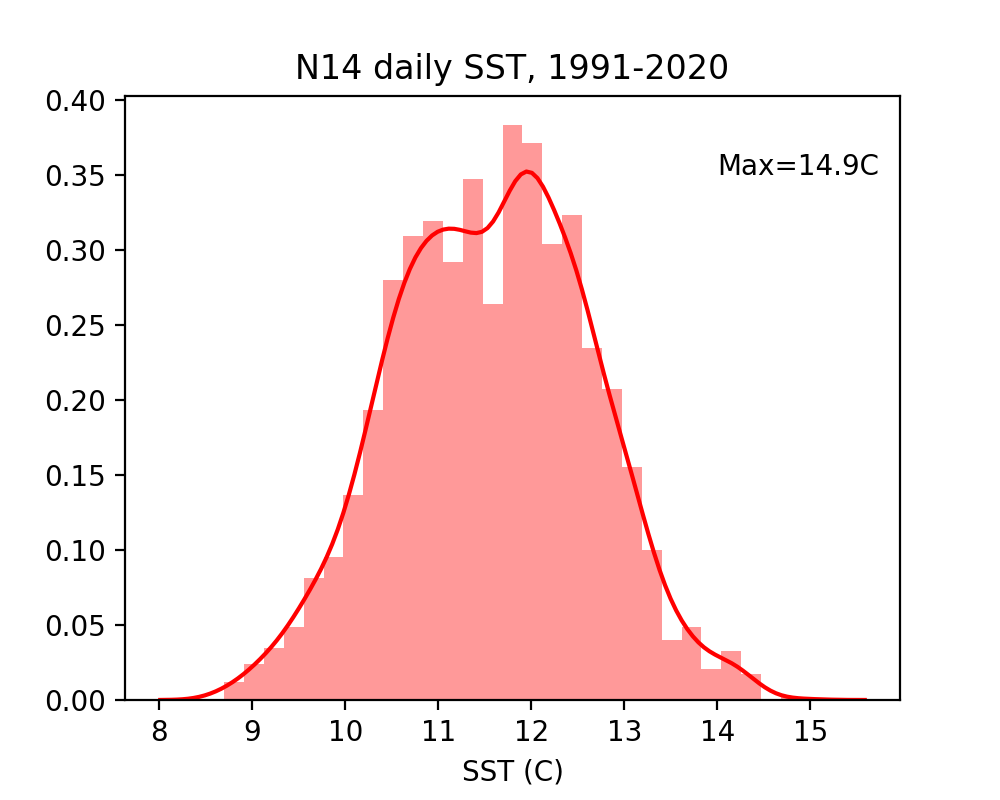

Supplement: S12 Fig — Daily SST values during winter (January-March) for buoy N14 for the period of study 1991–2020. (PNG) [file pone.0267737.s012.png]
