# Supplementary material for: Winter oceanographic conditions predict summer bull kelp canopy cover in northern California
Source: PLoS One. 2022 May 5;17(5):e0267737. doi: 10.1371/journal.pone.0267737 (PMC9070938; doi:10.1371/journal.pone.0267737)
Supplement: S1 Table — Only significant p<0.05 values shown (except BEUTI at 39°N), comparing two periods: pre-collapse (1991–2013), and entire period (1991–2020). Color indicates the sign of the trend. (PDF) [file pone.0267737.s013.pdf]

Winter trends	1991-2013	1991-2020
p-values <=0.05		
NorCal MOCI	0.019	-
CenCal MOCI	0.011	-
SoCal MOCI	0.032	-
BEUTI 41N	-	-
BEUTI 39N	0.051	-
BEUTI 37N	0.003	-
SST N14	0.015	-
SST N13	0.011	-

+	Positive trend
-	Negative trend
