# Supplementary material for: Winter oceanographic conditions predict summer bull kelp canopy cover in northern California
Source: PLoS One. 2022 May 5;17(5):e0267737. doi: 10.1371/journal.pone.0267737 (PMC9070938; doi:10.1371/journal.pone.0267737)
Supplement: S2 Table — 1 season lag in the first three columns and 2 seasons lag in the last column. Only statistically significant correlations p<0.05 are shown. (PDF) [file pone.0267737.s014.pdf]

	fall-1, winter	winter, spring	spring, summer	winter, summer
NorCal MOCI	-	0.68	-	-
CenCal MOCI	-	0.74	0.63	-
SoCal MOCI	-	0.45	0.78	0.37
BEUTI 41N	0.48	-	0.41	-
BEUTI 39N	0.54	-	-	-
BEUTI 37N	0.62	-	-	-
SSTN14	0.47	0.54	-	-
SSTN13	0.37	0.68	-	-
